# The crosstalk between efflux pump and resistance gene mutation in *Helicobacter pylori*

**DOI:** 10.1080/19490976.2024.2379439

**Published:** 2024-07-25

**Authors:** Xiaoling Gong, Youhua Wang, Ying An, Zhen Li, Dongsheng Liu, Xie Yong

**Affiliations:** a Department of Gastroenterology, Digestive Disease Hospital, The First Affiliated Hospital of Nanchang University, Nanchang, Jiangxi, China; b Department of Gastroenterology, Gastroenterology Institute of Jiangxi Province, Nanchang, Jiangxi, China; c Jiangxi Provincial Key Laboratory of Digestive Diseases, Department of Gastroenterology, The First Affiliated Hospital, Jiangxi Medical College, Nanchang, Jiangxi, China; d Department of Gastroenterology, Jiangxi Clinical Research Center for Gastroenterology, Nanchang, Jiangxi, China; e Department of Medical, Jinyu Medical Laboratory Co. Ltd, Shenyang Province, Liaoning, China; f Department of Clinical Nursing, Heze Health School, Shandong Province, Jinan, China

**Keywords:** *Helicobacter pylori*, efflux pump gene, resistance, crosstalk

## Abstract

Efflux pumps play a crucial role in the development of antibiotic resistance. The aim of this study was to investigate the relationship between efflux pump gene expression and resistance gene mutations in *Helicobacter pylori*. Twenty-six clinical strains with varying resistance characteristics were selected for further experiment. Seven susceptible strains were induced to become resistant, and the expression of efflux pump genes and point mutations were recorded. Four susceptible strains were selected to undergo candidate mutation construction, and changes in efflux pump gene expression were detected. Efflux pump knockout strains were constructed, and their effects on preventing and reversing antibiotic resistance gene mutations were assessed. Results showed that the expression of efflux pump genes hefA and hefD was significantly higher in the multidrug-resistant group compared to other groups. During the process of antibiotic-induced resistance, efflux pump gene expression did not exhibit a steady increase or decrease. Strains with the A2143G or A2142G point mutations in 23S rRNA exhibited lower hefA gene expression. Strains with mutations at 87K/91N, 87N/91 G, 87K/91D, or 87N/91Y in gyrA and the 194insertA mutation in rdxA showed higher hefA gene expression compared to the wild-type strain. During the process of antibiotic-induced resistance, the strain with the knockout of the efflux pump gene hefA developed mutations in the 23S rRNA, gyrA, or rdxA genes later compared to the wild-type strain. Knockout of the efflux pump gene could reverse the phenotypic resistance to clarithromycin or metronidazole in some strains but had no effect on reverse resistance gene mutation. This study suggested that different resistance gene point mutations may have varying effects on efflux pump gene expression. Knockout of the efflux pump gene can delay or prevent antibiotic resistance gene mutations to some extent and can reverse phenotypic resistance to clarithromycin and metronidazole in certain strains.

## Introduction

*Helicobacter pylori (H. pylori)* is a widespread human pathogen known for its pivotal role in gastrointestinal diseases.^1^ Eradication of *H. pylori* could significantly enhance patients’ quality of life and reduce the incidence of gastric cancer.^2^ However, the escalating rate of antibiotic resistance^3^ has led to a substantial decline in the efficacy of anti-*H. pylori* therapies, posing formidable challenges in treating *H. pylori* infections.^3^ Among the antibiotics commonly employed in anti-*H. pylori* therapy, clarithromycin (CLA), levofloxacin (LEV), and metronidazole (MET) exhibit notably high resistance rates. Our previous research indicates primary resistance rates of 22.1% for CLA, 19.2% for LEV, and a striking 78.2% for MET.^4^ Various mechanisms contribute to antibiotic resistance, including the production of drug-metabolizing enzymes that deactivate drugs^5–7^ conformational changes in drugs leading to their deactivation, alteration of drug binding sites, reduction in cell membrane permeability,^8^ and the activity of efflux pumps expelling intracellular drugs, such as antibiotics, that harm bacteria.^9^

Efflux pumps, responsible for expelling intracellular drugs from bacteria, play a pivotal role in the development of antibiotic resistance.^10^ Among the various types of efflux pumps identified in microorganisms, the resistance nodulation division (RND) family transporter stands out as the most significant in gram-negative bacteria.^11,12^ In *H. pylori*, there are three homologous genes clusters encoding proteins belonging to the RND family transporter, named hefA/B/C, hefD/E/F, hefG/H/I, respectively.^13^ Among these, hefA, hefD, and hefG encode outer membrane channel proteins and play pivotal roles in the efflux pump system.^13^ In recent years, several studies have focused on the relationship between efflux pump gene expression and antibiotic resistance, but a consensus has not been reached in the literature. It was believed that the expression of efflux pump was primarily associated with the emergence of multidrug resistance.^10,14^ However, some researchers have observed high expression of the efflux pump gene hefA in MET-resistant strains and amoxicillin-resistant strains.^5,15^ Furthermore, the use of efflux pump inhibitors or the knockout of the efflux pump gene hefA has been shown to increase the sensitivity of certain strains to MET, CLA and amoxicillin.^14,16^ Conversely, re-introducing the hefA gene after its knockout has been found to reduce the sensitivity to MET and amoxicillin once again. However, in some other investigations, the knockout of the efflux pump gene hefC showed no significant effect on changes in antibiotic sensitivity.^17^ Furthermore, is there any association between the expression of efflux pump genes and mutations in antibiotic resistance genes? Lee SM discovered that knockout of hefA failed to reverse mutations in rdxA and frxA observed in resistant isolates, even after their transfer to susceptible isolates subsequent to hefA knockout.^15^ There has been a lack of research attention directed toward the impact of resistance gene mutations on efflux pump gene expression.

To elucidate the functions of the RND family genes hefA, hefD, and hefG in the process of antibiotic resistance production in *H. pylori*, as well as to investigate any potential relationships between efflux pump gene expression and specific resistance gene mutations, we conducted the present experiment. Furthermore, we aimed to explore the possibility of preventing the occurrence of specific gene mutations through the knockout of efflux pump genes.

## Method

### Bacterial

The *H. pylori* standard strain 26,695 was kind gifts from Sun Y-d (Shandong University, Shandong province, China) and eight-hundred clinical *H. pylori* strains were obtained and stored at The First Affiliated Hospital of Nanchang University, Jiangxi, China. After screening, twenty-six clinical strains were included, eight clinical *H. pylori* isolates susceptible to CLA, LEV, and MET (named J5A, J12, 18, R28, R32, 39, J59 and R96), five clinical *H. pylori* isolates resistant to CLA, LEV, and MET (named M10MDR, F38MDR, 56MDR, 92MDR and 189MDR), six clinical *H. pylori* isolates susceptible to CLA and LEV but resistant to MET (named 9MZ, 21MZ, 23MZ, 99MZ, 305MZ, 409MZ), five clinical *H. pylori* isolates susceptible to CLA and MET but resistant to LEV (named 298LE, 319LE, 498LE, 632LE, 639LE), and two clinical *H. pylori* isolates susceptible to LEV and MET but resistant to CLA (named 100CH and 184CH). *H. pylori* strains were initially cultured on Campylobacter agar (Oxoid, Basingstoke, UK) plates supplemented with 5% defibrinated sheep blood (Bio-Kont, Zhejiang, China). The plates were incubated in a microaerobic atmosphere (10% CO2, 5% O2, and 85% N2) at 37°C for 3day.

### Antimicrobial susceptibility testing

Susceptibility testing for CLA, LEV, and MET were conducted using the E-test method. The resistance breakpoint for CLA, LEV, and MET were set at >0.5 mg/L, > 1 mg/L and >4 mg/L, respectively, which were selected using breakpoint tables for the interpretation of MICs (minimum inhibitory concentration) provided by the European Committee on Antimicrobial Susceptibility Testing Version 9.0, 2019 (http://www.eucast.org.) *H. pylori* culture and antibiotic susceptibility testing were performed by the Institute of Gastroenterology and Hepatology of the First Affiliated Hospital of Nanchang University.

The agar dilution method was used obtained the original MICs of CLA, LEV, and MET in these seven clinical Susceptible *H. pylori* strains and standard strain 26,695 that selected for further Antibiotic-resistant strain development experiment. For agar dilution, the agents CLA, LEV, and MET were obtained from the China Institute for Food and Drug control as standard powders with known potencies. All compounds were dissolved and diluted according to the manufacturers’ recommendations, and solutions were used on the day of preparation. The plates contained twofold dilutions of antibiotics with concentrations ranging from 0.002 to 256 mg/L for CLA, from 0.002 to 32 mg/L for LEV and from 0.008 to 256 mg/L for MET.

## Genomic DNA extraction, sequencing and mutation analysis

DNA extraction from strains was conducted using a Tiangen DNA Mini Kit (Tiangen, Beijing, China) according to the manufacturer’s instructions. Polymerase chain reaction (PCR) amplification of 23S rRNA, gyrA and rdxA was performed with the following primers. The primer sequences used were as follows: 23S rRNA_F (5’-CCACAGCGATGTGGTCTCAG-3’), 23S rRNA_R (5’-CTCCATAAGAGCCAAAGCCC-3’), (product 425bp); gyrA_F (5’-AGCTTATTCCATGAGCGTGA-3’), gyrA _R (5’-TCAGGCCCTTTGACAAATTC-3’), (product 582bp); rdxA_F (5’- TTACAGAGAGCCAGA TAGCC-3’), rdxA _R (5’-CACAA -CCAAGTAATCGCATC-3’), (product 780bp). PCR amplicons were sent to Sangon Biotech (Shanghai, China) for Sanger sequencing. The DNA sequence data were analyzed using the DNAMAN software (2005, Lynnon) following alignment with a reference sequence (*H. pylori* 26695).

## Gene expression analysis

*H. pylori* strains were retrieved from Columbia agar plates, and their concentrations were adjusted to an optical density (OD) of 0.2 in Brucella broth medium supplemented with 5% fetal bovine serum (FBS). The bacterial suspension was cultured overnight at 37°C with shaking under microaerobic conditions. Total mRNA of *H. pylori* strain was isolated from the bacterial suspension using RNAiso Plus (Takara, Dalian, China). mRNA was reverse transcribed into cDNA using the FastKing cDNA reagent kit (Tiangen, Beijing, China) according to the manufacturer’s instructions. Quantitative PCR amplification of hefA, hefD, hefG and 16SrRNA was performed with the following primers: hefA_F (5’- TATGCCCGCTGTTGA −3’), hefA_R (5’- GAGGAAATACGACGCTAA −3’), (product 143bp); hefD_F (5’- ATTCGGGATTGGCTC-3’), hefD_R (5’- TAGGAGTTGGCGTTGA −3’), (product 180bp); hefG_F (5’- CATTTGAGATTGCGTGA −3’), hefG_R (5’- TCGTTAGCAAGTGGGATA-3’), (product 158bp); 16SrRNA_F (5’- GGAGTACGGTCGCAGATTAAA −3’), 16SrRNA _R (5’- CTAGCGGATTCTCTCAATGTCAA −3’), (product 127bp).

## Antibiotic-resistant strain development

Susceptible *H. pylori* strains were inoculated onto Columbia agar plates, and the agar dilution method was employed to determine the original MIC (MICori) values for each strain. Subsequently, the *H. pylori* strains were stimulated with CLA/LEV/MET to induce antibiotic resistance individually. The process involved the following steps: *H. pylori* strains were retrieved from Columbia agar plates, and their concentrations were adjusted to an optical density (OD) of 0.2 in Brucella broth medium supplemented with 5% FBS, the bacterial suspension was cultured overnight at 37°C with shaking under microaerobic conditions. Antibiotics were added to Brucella broth medium to achieve a concentration of 1/2 MICori. After culturing for 6 hours, the medium was transferred to Columbia agar plates containing 1/2 MICori and incubated for 2–3 days. Single colonies grown on these plates were designated as Hp_1/2×MICori and selected for the subsequent step of resistance development. This process was repeated to obtain Hp_1×MICori, Hp_2×MICori, Hp_4×MICori, and so forth, with the antibiotic concentration progressively increasing. The process was terminated when the antibiotic concentration reached 32 mg/L or when resistance induction failed three times.

## Natural transformation of the candidate mutation

The amplified PCR products containing either wild-type sequences or candidate mutations were separately introduced into four susceptible strains through natural transformation, as previously described. Recipient cells were inoculated onto Mueller-Hinton agar plates and allowed to grow for 6 hours. Subsequently, PCR fragments diluted in TE (10 mM Tris-HCl [pH 8.0] and 1 mM EDTA) was added directly onto the bacterial lawn. After incubation for 24 h under microaerophilic conditions, the transformed cells were streaked onto Mueller-Hinton II agar plates containing CLA/LEV/MET at concentrations ranging from 0.25 to 32 mg/liter. Several single colonies from the lowest to the highest concentrations on the antibiotic-containing plates were spread onto antibiotic-free defibrinated sheep blood agar plates. Successful transformations and mutations were confirmed with PCR, followed by DNA sequencing analysis.

## Construction of *H. pylori* mutants

The plasmids pILL570 and pUC18K2 were generously provided by Sun Y-d (Shandong University, Shandong province, China). The construction of hefA, hefD, and hefG mutant strains was identical to the construction of the knockout strain (Δstrain), as detailed in the literature.^18^ Briefly, the genes hefA, hefD, and hefG from the genome of *H. pylori* 26,695 were disrupted by inserting the nonpolar aphA-3 gene, which encodes a kanamycin resistance cassette. All primers utilized in these studies are listed as follows: hefA_UF (5'-GCTGCAGCGGGGTTACTGCGACTTTG-3'), hefA_UR (5'-GGAATTCCGCTTGGAGTTGTTGGGTG-3'); hefA_DF (5'-CGGATCCGGGGGTTACTGCGACTTTG-3'), hefA_DR (5'-CATCGATGACTCGCTATCACGCCATC-3'); hefD_UF (5'-GCTGCAGCACGCCTAGCCAATATCAA-3'), hefD_UR (5'-GGAATTCCAAAGTTCGCTTCTCGTTG-3'); hefD_DF (5’-CGGATCCGTCAACGCCAACTCCTACT-3'), hefD_DR (5'-CATCGATGGCTCGTTTGATACTCCCT-3'); hefG_UF (5'-GCTGCAGCTTTATGATGCCTGAAATGC-3'), hefG_UR (5'-GGAATTCCATTAAATTGGGCGAACTG-3'); hefG_DF (5'-CGGATCCGAAATCGCACAAATTTATG-3'), hefG_DR (5'-CATCGATGATCCCTTCTACAAACAAGAT-3'). The hefA/hefD/hefG gene of *H. pylori* strain 26695 was amplified using specific primers and cloned into the pMD18T-vector (Promega), resulting in the plasmid pMD18T-hefA/hefD/hefG. Subsequently, the hefA/hefD/hefG gene was disrupted by inserting the kanamycin resistance gene from pAV35 into a unique EcoRV restriction site, generating the plasmid pILL570. This plasmid was then introduced into E. coli ER1793 and utilized for natural transformation of H. pylori, as described previously. Colonies derived from two independent transformations were examined. Correct allelic replacement of the wild-type gene with the interrupted version was confirmed using PCR.

## Statistical analysis

All statistical analyses were conducted using SPSS Statistics for Windows (version 21.0, IBM Corp, Armonk, NY, USA). Chi-square and Fisher’s exact tests were employed to assess the statistical significance of differences between categorical variables. A p-value of ≤0.05 was considered statistically significant.

## Results

### The association between efflux pump genes and antimicrobial susceptibility

**Figure 1.**
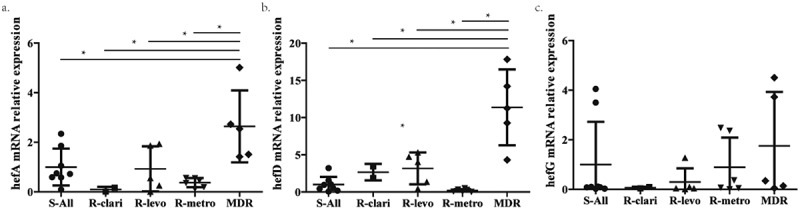
mRNA level of efflux pump gene in different clinical strains. a. The mRNA level of efflux pump gene hefA in susceptible strain (S-All), clarithromycin resistant strain (R-clari), levofloxacin resistant strain (R-levo), metronidazole resistant strain (R-metro) and multiple resistant strain (MDR). b. The mRNA level of efflux pump gene hefD in susceptible strain, clarithromycin resistant strain, levofloxacin resistant strain, metronidazole strain and multiple resistant strain. c. The mRNA level of efflux pump gene hefG in susceptible strain, clarithromycin resistant strain, levofloxacin resistant strain, metronidazole strain and multiple resistant strain. *: *p* < 0.05.

After screening, twenty-six strains were selected, comprising eight clinical *H. pylori* isolates susceptible to CLA, LEV, and MET (S-All strain), five clinical *H. pylori* isolates resistant to CLA, LEV, and MET (MDR strain), six clinical *H. pylori* isolates susceptible to CLA and LEV but resistant to MET ((R-metro strain)), five clinical *H. pylori* isolates susceptible to CLA and MET but resistant to LEV (R-levo strain), and two clinical *H. pylori* isolates susceptible to LEV and MET but resistant to CLA (R-clari strain). The mRNA levels of genes hefA, hefD, and hefG were compared among the different groups. The results demonstrated that hefA and hefD expression levels were significantly higher in the multidrug-resistant group than in the other groups (*p* < 0.05). However, the mRNA expression of hefG did not show significant differences among the different groups (Figure 1).

### The association of antibiotic resistance gene mutation and antimicrobial susceptibility

The genotype characteristics of these resistant strains were further elucidated using gene sequencing methods. For CLA resistant strain, a single A2143G point mutation in the 23S rRNA was identified in five MDR strains and two R-clari strains (Table 1). Regarding LEV resistant strains, various gyrA amino acid mutants including 87I/91D, 87K/91D, 87K/91 G, 87N/91 G, 87N/91N, 87N/91D and 87N/91Y were detected in five MDR strains and five R-levo strains (Table 1). As for MET resistant strain, point mutations in the rdxA gene posed challenges, with their abundance observed in five MDR strains and six R-metro strains, yet their repeatability was notably poor (Table 1).

**Table 1.** Genotype characteristic of resistant strains.

gene	M10MDR	F38MDR		56MDR	92MDR	189MDR	100CH	184CH	298LE	319LE
23S rRNA	A21G3G	A21G3G		A21G3G	A21G3G	A21G3G	A21G3G	A21G3G	–	–
gyrA	87I/91D	87K/91D		87N/91N	87N/91 G	87K/91D	–	–	87N/91 G	87N/91D
rdxA	M56I, A80T, A108S, S118A, Q119del	Q50del, R53H, A108S, S118A, I172V		41insertEIA, V62L, A108S, S118A	R53H, S118A,	A108S, S118A,	–	–	–	–
gene	498LE		632LE	639LE	9MZ	21MZ	23MZ	99MZ	305MZ	409MZ
23S rRNA	–		–	–	–	–	–	–	–	–
gyrA	87N/91 G		87I/91D	87N/91Y	–	–	–	–	–	–
rdxA	–		–	–	A108S, S118A	A108S, S118A	M21I, V62L, A80V, M84I, A108S, S118A	S43L, V62L, A108S, S118A	S118A	A108S, S118A

MDR, multiple resistant strain; R-clari, resistance for clarithromycin; R-Levol, resistance for levofloxacin; R-Metrol, resistance for metronidazole; A2143G, original G base at position 2143 is replaced by the A base; 87I/91D, The amino acid sequence of the 87th position of the gyrA gene is I and the 91th position of the gyrA gene is D; (I: Isoleucine, D: Aspartic acid, K: Lysine, N: Asparagine, G: Glycine, Y: Tyrosine, M: Methionine, T: Threonine, Q: Glutamine, del, R: Arginine, H: Histidine, insertEIA: insert Glutamate, Isoleucine and Alanine, V: Valine, L: Leucine, A: Alanine).

### Changes in efflux pump gene expression and antibiotic resistance gene mutation during the process of antibiotic-induced resistance

Seven susceptible strains were induced to develop resistance to CLA, LEV, and MET based on their MICori values (Table 2), respectively.

**Table 2.** Origin MIC of susceptible strains for further antibiotic-resistant strain development.

Strain	MICori			1/2 MICori		
	CLA(mg/L)	LEV(mg/L)	MET(mg/L)	CLA(mg/L)	LEV(mg/L)	MET(mg/L)
J5A	0.031	0.25	1	0.016	0.125	0.5
J12	0.031	0.5	1	0.016	0.25	0.5
R28	0.016	0.031	1	0.008	0.016	0.5
R32	0.008	0.25	1	0.004	0.125	0.5
39	0.008	0.25	4	0.004	0.125	2
J59	0.008	0.5	0.5	0.004	0.25	0.25
R96	0.008	0.5	0.5	0.004	0.25	0.25

### Changes in efflux pump gene expression and antibiotic resistance gene mutation during the process of CLA-induced resistance

Seven susceptible clinical strains were selected to induced resistance with CLA as described in the methods section. Among these, six strains successfully developed resistance, while one strain failed to develop CLA resistance after three attempts.

**Figure 2.**
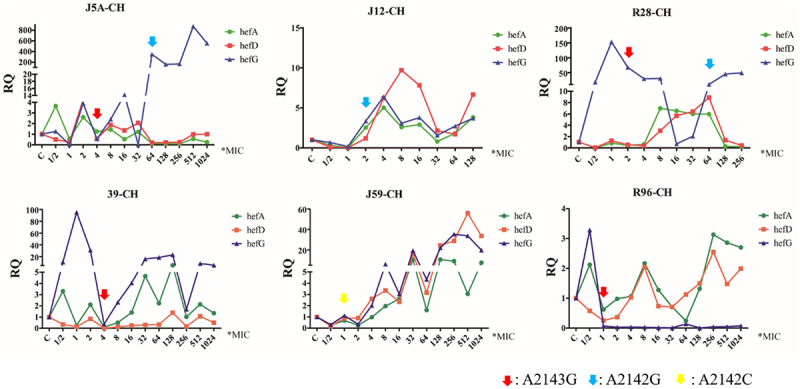
Changes in efflux pump gene expression and 23S rRNA gene mutation during the process of clarithromycin-induced resistance (RQ: relative quantitative mRNA expression, CH: clarithromycin; different arrows represent different point mutations).

During the induction process, changes in efflux pump gene expression and point mutations were monitored. The results revealed that the expression of efflux pump genes hefA, hefD, and hefG did not exhibit a consistent pattern of increase or decrease. Mutations in the 23S rRNA gene were observed after exposure to high concentrations of antibiotics (above MICori). Specifically, A2143G, A2142G, and A2142C point mutations were detected in these strains. Interestingly, a decrease in hefA and hefD expression was noted when A2143G mutations were detected. Additionally, when A2142G mutations occurred after A2143G, a significant increase in hefG expression was observed. A2142C mutations were only detected in one strain, and the expression levels of hefA, hefD, and hefG did not exhibit substantial changes (Figure 2).

### Changes in efflux pump gene expression and antibiotic resistance gene mutation during the process of LEV-induced resistance

**Figure 3.**
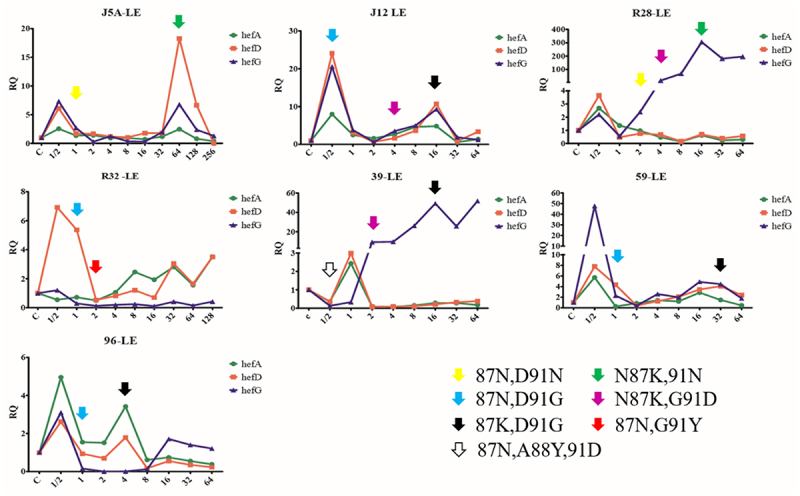
Changes in efflux pump gene expression and gyrA gene mutation during the process of levofloxacin-induced resistance (LE: levofloxacin, different arrows mean different amino acid mutations (N: asparagine, D: aspartic acid, K: lysine, G: glycine, Y: tyrosine, A: alanine).

All seven susceptible strains successfully developed resistance to LEV. Changes in efflux pump gene expression and point mutations were recorded during the process. The results indicate that the expression of efflux pump genes hefA, hefD and hefG did not exhibit a consistent pattern of increase or decrease. GyrA mutations were observed in five strains when exposed to high concentrations of antibiotics (above MICori), while two strains exhibited mutations when stimulated with a concentration of 1/2 MICori. Mutations at the 91st aspartic acid and the 87th aspartic amide in the gyrA gene were detected in all seven induction series. Specifically, the mutation at the 91st aspartic acid was the first mutation detected in the induction process of six strains, while the mutation at the 88th alanine was the first mutation detected in one strain. Moreover, mutations at the 91st amino acid could be reversed when the 87th aspartic amide mutated (Figure 3).

### Changes in efflux pump gene expression and antibiotic resistance gene mutation during the process of MET-induced resistance

**Figure 4.**
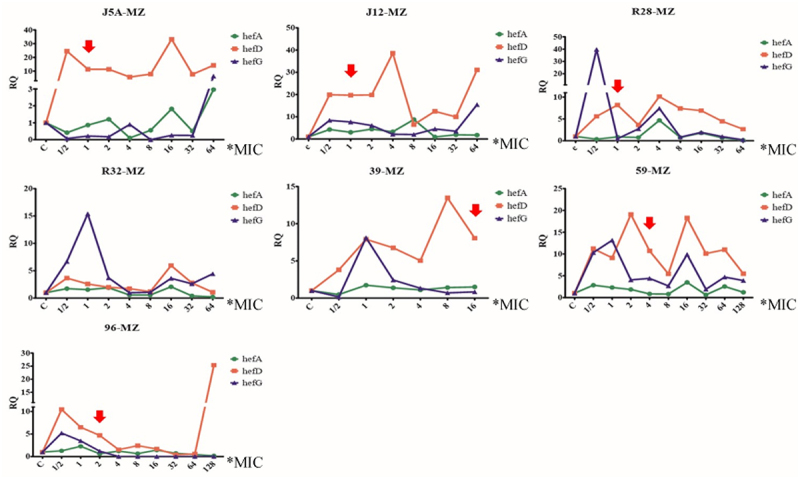
Changes in efflux pump gene expression and rdxA gene mutation during the process of metronidazole-induced resistance (red arrow means the first amino acid mutation, MZ: metronidazole).

All seven susceptible strains successfully developed resistance to MET. Changes in efflux pump gene expression and point mutations were recorded during the process. The results revealed that the expression of efflux pump genes hefA, hefD, and hefG did not exhibit a consistent pattern of increase or decrease. While one strain did not show any mutations during the induction of resistance, the other six strains exhibited mutations (Figure 4); however, their repeatability was poor (Table 3).

**Table 3.** Characteristics of rdxA mutation during the process of metronidazole-induced resistance.

Strain	First mutation	Second mutation	Third mutation
J5A	S29F, S43F, T58I	Frameshift(75insertA)	
J12	Frameshift (194 insertA)	T67S, K73R, T79S, K81N, K127R	-
R28	E133K,I172N	E75K,E138K,N172I	K75E,K138E,I172N
R32	–	–	–
39	E133K	–	–
J59	R16C	C16H,E73G,N98S,A118S,S150G	–
R96	N84K,E74G,	H17Y,P51T,G74E	–

## The impact of antibiotic resistance gene mutation on efflux pump gene expression

### The impact of 23SrRNA gene mutation on efflux pump gene expression

**Figure 5.**
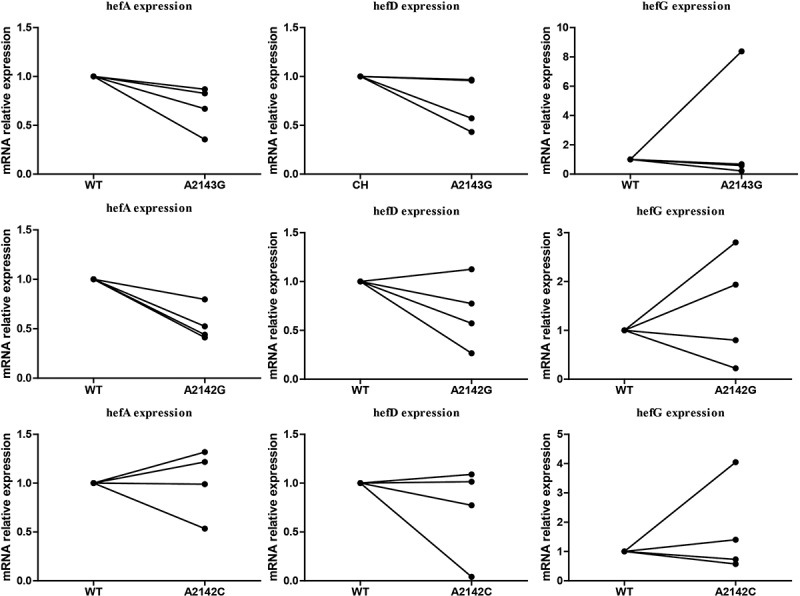
Comparison of efflux pump gene expression (hefA/hefD/hefG) between wild-type strain and 23S rRNA mutant strains.

During the process of CLA gradient induction resistance, three different mutations (A2142C, A2142G, A2143G) in the 23S rRNA gene were obtained. These mutations were then transferred into four susceptible *H. pylori* strains (including three clinical susceptible strains (J5A, R28 and 39) and the standard strain 26,695) to construct CLA resistance mutant strains. Subsequently, the expression of efflux pump genes was detected. The results indicated that strains with A2143G and A2142G point mutations exhibited lower hefA gene expression compared to the wild-type strain (Figure 5).

### The impact of gyrA gene mutation on efflux pump gene expression

**Figure 6.**
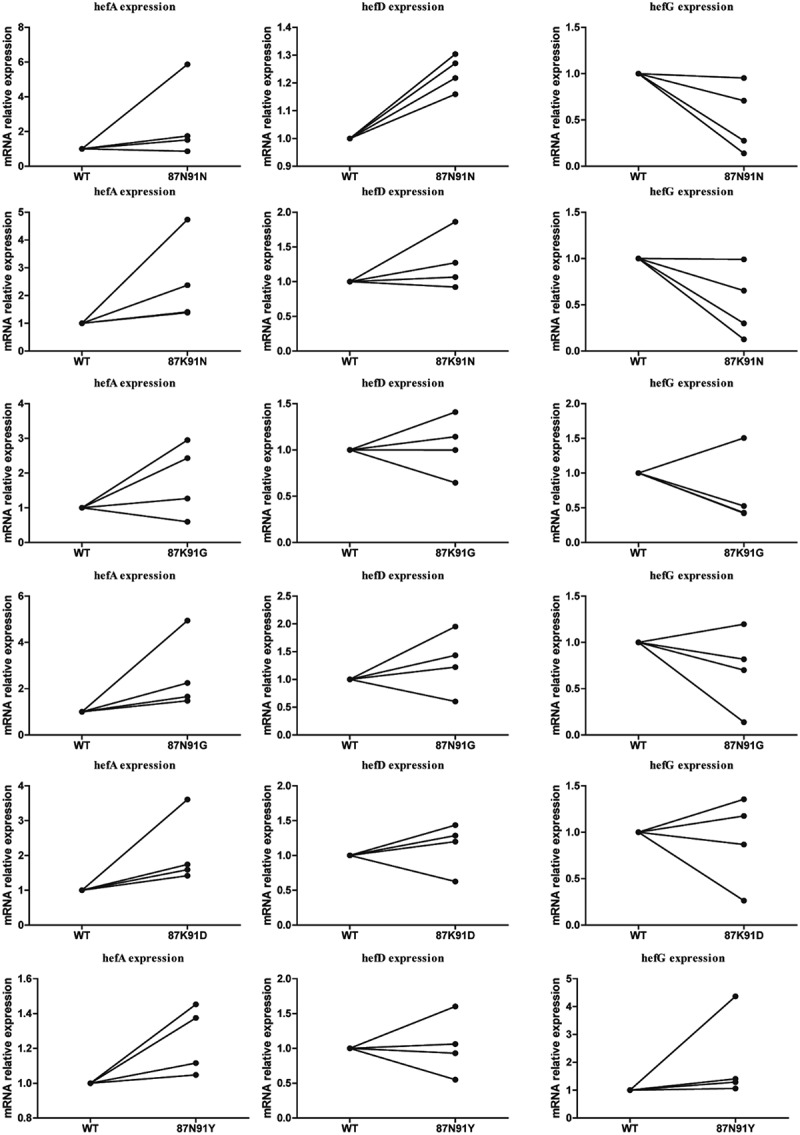
Comparison of efflux pump gene expression between wild-type strain and gyrA mutant strains.

During the LEV gradient induction resistance process, six different amino acid mutations (87N/91N, 87K/91N, 87K/91 G, 87N/91 G, 87K/91D, and 87N/91Y) in the gyrA gene were obtained. These mutations were then transferred into four strains (including three clinical susceptible strains (J5A, R28 and 39) and the standard strain 26,695) to construct LEV-resistant mutant strains. Subsequently, the expression of efflux pump genes was recorded. The results indicated that strains with 87K/91N, 87N/91 G, 87K/91D, and 87N/91Y mutations exhibited higher hefA gene expression compared to the wild-type strain. Additionally, the strain with 87N/91N mutation demonstrated higher hefD gene expression and lower hefG gene expression (Figure 6).

### The impact of rdxA gene mutation on efflux pump gene expression

**Figure 7.**
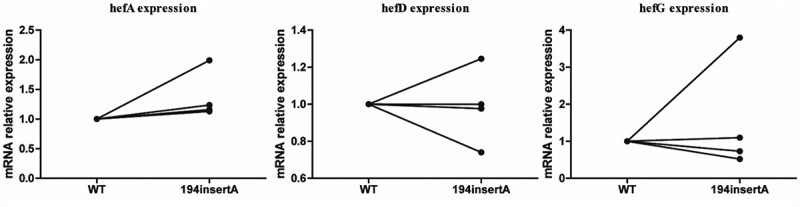
Comparison of efflux pump gene expression between wild-type strain and rdxA mutant strains.

During the MET gradient induction resistance process, three different amino acid mutations of the rdxA gene were obtained. These mutations were transferred into four strains (including three clinical susceptible strains (J5A, R28 and 39) and the standard strain 26,695) to construct MET-resistant mutant strains. However, only the mutation of rdxA gene 194insertA was successfully constructed in all four recipient strains. The remaining two amino acid mutations failed to be screened as single mutations were hard to maintained. The results revealed that the strain with the 194insertA mutation exhibited higher hefA gene expression compared to the wild-type strain (Figure 7).

## The role of efflux pump genes in preventing antibiotic resistance gene mutations

The standard strain 26,695 and its mutant strain 26695_ΔhefA, 26695_ΔhefD, and 26695_ΔhefG were selected to develop antibiotic resistance with CLA, LEV, and MET respectively. The DNA of each strain was collected and sequenced step by step.

### Efflux pump gene knockout delays 23SrRNA gene mutation

**Figure 8.**
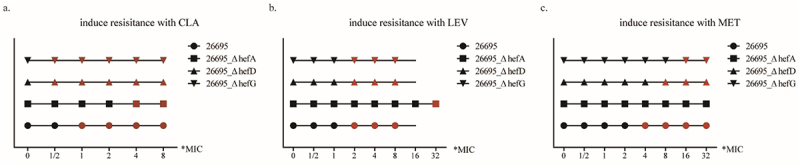
The effect of efflux pump gene in prevent antibiotic resistance gene mutations (black point indicates similarity to the original sequence, while red point indicates mutation in the target gene).

The results revealed that during induction with CLA, strain 26,695 exhibited 23S rRNA gene A2142C mutations at the 1*MIC induction stage. Strain 26695_ΔhefA showed 23S rRNA gene A2143G mutations at the 4*MIC induction stage. Strains 26695_ΔhefD and 26695_ΔhefG exhibited 23S rRNA gene A2143G mutations at the 1/2*MIC induction stage (Figure 8(a)). These findings indicate that the knockout of the efflux pump gene hefA can delay the occurrence of 23S rRNA gene mutations.

### Efflux pump gene knockout delays gyrA gene mutation

When induced with LEV, strain 26,695 exhibited gyrA gene 272 base mutations at the 2*MIC induction stage. Strain 26695_ΔhefA showed gyrA gene 272 base mutations at the 32*MIC induction stage. Strains 26695_ΔhefD and 26695_ΔhefG exhibited gyrA gene 273 base mutations at the 2*MIC induction stage (Figure 8(b)). These results demonstrate that the knockout of the efflux pump gene hefA can delay the occurrence of gyrA gene mutations.

### Efflux pump gene knockout delays or prevents rdxA gene mutation

During induction with MET, strain 26,695 exhibited rdxA gene 146 base mutations at the 4*MIC induction stage. Strain 26695_ΔhefA did not show any rdxA gene base mutations throughout the entire induction stage. Strain 26695_ΔhefD exhibited rdxA gene 146 base mutations at the 8*MIC induction stage, while strain 26695_ΔhefG exhibited rdxA gene 223 base mutations at the 16*MIC induction stage (Figure 8(c)). These results indicate that the knockout of the efflux pump genes hefA, hefD, and hefG can delay or prevent rdxA gene mutations from occurring.

## Efflux pump gene knockout reverses antibiotic resistance gene mutation

Several MDR strains and mono-resistant strains were selected construct their mutant strain, strain_ΔhefA, strain_ΔhefD and strain_ΔhefG, respectively.

The MIC of CLA was detected for the original strains and mutant strains. The results indicate that compared to the original strains 184CH and 100CH, their mutant strains 184CH_ΔhefA, 100CH_ΔhefA, 100CH_ΔhefD, and 100CH_ΔhefG exhibited susceptibility to CLA. Although three MDR strains showed a decrease in the MIC value of CLA, they remained resistant (Table 4). Additionally, we investigated the changes in the 23SrRNA gene mutation after efflux pump gene knockout. The results showed that the 23SrRNA gene remained consistent in both the original strains and mutant strains. These findings suggest that knockout of efflux pump genes can reverse CLA resistance in some strains but has no effect on 23SrRNA gene mutation.

**Table 4.** Efflux pump gene knockout reverses antibiotic resistance.

	MIC for clarithromycin							MIC for levofloxacin						
strain	100CH (mg/L)		184CH (mg/L)	F38MDR (mg/L)		M10MDR (mg/L)	92MDR (mg/L)	639LE (mg/L)		298LE (mg/L)	F38MDR (mg/L)		M10MDR(mg/L)	92MDR(mg/L)
Ori	6		12	256		32	256	8		2	32		32	32
ΔhefA	0.5		0.75	2		2	256	2		2	8		12	32
ΔhefD	0.75		1.5	256		2	256	6		2	32		32	32
ΔhefG	0.25		8	256		2	256	6		2	8		16	32
		MIC for metronidazole												
strain		23MZ(mg/L)			F38MDR(mg/L)				M10MDR(mg/L)			92MDR(mg/L)		
Ori		48			256				256			256		
ΔhefA		0.38			256				0.5			256		
ΔhefD		6			256				0.38			256		
ΔhefG		32			256				0.38			256		

Change of MIC for clarithromycin/levofloxacin/metronidazole between wild-type strain and efflux pump gene knockout strain. ori: original MIC.

The MIC of LEV was determined for both the original strains and mutant strains. The results indicated that several mono-LEV resistant strains and MDR strains exhibited a decrease in the MIC value of LEV, although none of the strains reverted to a susceptible status (Table 4). Additionally, we investigated the changes in gyrA amino acid mutation after efflux pump gene knockout. However, the gyrA gene remained consistent in both the original strains and mutant strains. These findings suggest that knockout of efflux pump genes cannot reverse LEV resistance and has no effect on gyrA amino acid mutation.

The MIC of MET was determined for both the original strains and mutant strains. The results revealed that compared to the original strains M10MDR and 23MZ, their mutant strains M10MDR_ΔhefA, M10MDR_ΔhefD, M10MDR_ΔhefG, 23MZ_ΔhefA, and 23MZ_ΔhefD exhibited susceptibility to MET. However, other strains showed either a mild decrease or no change in the MIC value of MET, maintaining their resistance status (Table 4). Additionally, we investigated the changes in rdxA gene mutation after efflux pump gene knockout. Several rdxA gene mutations were detected in both the original strains and the mutant strains, although their repeatability was poor. These findings suggest that knockout of efflux pump genes can reverse MET resistance in some strains.

## Discussion

Efflux pumps, responsible for pumping intracellular drugs out of bacteria, play a crucial role in the development of antibiotic resistance. Despite numerous studies focusing on the relationship between efflux pump gene expression and antibiotic resistance, a consensus has yet to be reached. In this study, we observed higher expression of efflux pump genes hefA and hefD in MDR strains, while mono-resistant strains did not exhibit these changes. The genotypic characteristics of these resistant strains were found to be varied for gyrA and rdxA genes, prompting an exploration into whether different genotypes would have varying effects on efflux pump gene expression.

To investigate this, we induced antibiotic resistance in susceptible strains and observed that efflux pump gene expression did not consistently increase or decrease during the process. Notably, *H. pylori* strains with A2143G or A2142G point mutations in 23S rRNA exhibited lower hefA gene expression. Additionally, strains with specific mutations (87K/91N, 87N/91 G, 87K/91D, or 87N/91Y) in gyrA demonstrated higher hefA gene expression compared to the wild-type strain. Strains with the 87N/91N mutation in gyrA exhibited higher hefD gene expression but lower hefG gene expression. Efforts to construct several rdxA point mutation strains resulted in only one successful mutation. Interestingly, the strain with the 194insertA mutation in rdxA exhibited higher hefA gene expression. These findings suggest that different point mutations may have varying effects on efflux pump gene expression.

Some research suggests that using efflux pump inhibitors or knocking out efflux pump genes could increase the sensitivity of certain antibiotics like MET and amoxicillin. To explore the effect of efflux pump genes on resistance gene mutation, we selected susceptible standard strains to construct efflux pump knockout strains and induced resistance with CLA, LEV, and MET, respectively.

The results indicated that knocking out the efflux pump gene hefA could delay 23S rRNA and gyrA gene mutations and could also delay or prevent rdxA gene mutations in some *H. pylori* strains. Conversely, knocking out the efflux pump genes hefD and hefG did not exhibit this effect.

Furthermore, we selected MDR and mono-resistant strains to construct efflux pump gene knockout strains. The results demonstrated that knocking out efflux pump genes could reverse CLA resistance in some strains but had no effect on 23S rRNA gene mutations. Additionally, knocking out efflux pump genes did not reverse LEV resistance and had no effect on gyrA gene mutations.

## Conclusion

Efflux pump genes play a crucial role in antibiotic resistance. Different point mutations may have varying effects on efflux pump genes expression. Knocking out efflux pump genes can delay or prevent antibiotic resistance gene mutations to some extent and can even reverse the resistance to CLA and MET in certain strains.

## Data Availability

All data generated or analyzed during this study are included in this published article and its supplementary information files.

## References

[1] Moss SF, Shah SC, Tan MC, El-Serag HB. Evolving concepts in Helicobacter pylori management. Gastroenterology. 2024;166(2):267–14. doi:10.1053/j.gastro.2023.09.047. 37806461 PMC10843279

[2] Ford AC, Forman D, Hunt RH, Yuan Y, Moayyedi P. Helicobacter pylori eradication therapy to prevent gastric cancer in healthy asymptomatic infected individuals: systematic review and meta-analysis of randomised controlled trials. BMJ. 2014;348:g3174. doi:10.1136/bmj.g3174. 24846275 PMC4027797

[3] Kuo YT, Liou JM, El-Omar EM, Wu JY, Leow A, Goh KL, Das R, Lu H, Lin JT, Tu YK. et al. Primary antibiotic resistance in Helicobacter pylori in the Asia-Pacific region: a systematic review and meta-analysis. Lancet Gastroenterol Hepatol. 2017;2(10):707–715. doi:10.1016/S2468-1253(17)30219-4. 28781119

[4] S LD, H WY, R ZZ, Y ZZ, Lu H, M XJ, Du YQ, Li Y, B WJ, P XS. et al. Primary antibiotic resistance of Helicobacter pylori in Chinese patients: a multiregion prospective 7-year study. Clin Microbiol Infect. 2018;24(7):780–785. doi:10.1016/j.cmi.2017.11.010. 29138101

[5] Rimbara E, Mori S, Kim H, Suzuki M, Shibayama K. Mutations in genes encoding penicillin-binding proteins and efflux pumps play a role in β-lactam resistance in helicobacter cinaedi. Antimicrob Agents Chemother. 2018;62(2). doi:10.1128/AAC.02036-17. PMC5786776 29203490

[6] Kuo CJ, Ke JN, Kuo T, Lin CY, Hsieh SY, Chiu YF, Wu HY, Huang MZ, Bui NN, Chiu CH. et al. Multiple amino acid substitutions in penicillin-binding protein-1A confer amoxicillin resistance in refractory Helicobacter pylori infection. J Microbiol Immunol Infect. 2023;56(1):40–47. doi:10.1016/j.jmii.2022.07.006. 35995672

[7] Attaran B, Salehi N, Ghadiri B, Esmaeili M, Kalateh S, Tashakoripour M, Eshagh HM, Mohammadi M. The penicillin binding protein 1A of Helicobacter pylori, its amoxicillin binding site and access routes. Gut Pathog. 2021;13(1):43. doi:10.1186/s13099-021-00438-0. 34183046 PMC8240269

[8] Tang X, Yang T, Shen Y, Song X, Benghezal M, Marshall BJ, Tang H, Li H. Roles of lipopolysaccharide glycosyltransferases in maintenance of Helicobacter pylori morphology, cell wall permeability, and antimicrobial susceptibilities. Int J Mol Sci. 2023;24(14). doi:10.3390/ijms241411381. PMC10379358 37511140

[9] Cai Y, Wang C, Chen Z, Xu Z, Li H, Li W, Sun Y. Transporters HP0939, HP0497, and HP0471 participate in intrinsic multidrug resistance and biofilm formation in Helicobacter pylori by enhancing drug efflux. Helicobacter. 2020;25(4):e12715. doi:10.1111/hel.12715. 32548895

[10] Liu ZQ, Zheng PY, Yang PC. Efflux pump gene hefA of Helicobacter pylori plays an important role in multidrug resistance. World J Gastroenterol. 2008;14(33):5217–5222. doi:10.3748/wjg.14.5217. 18777600 PMC2744013

[11] Mehrabadi JF, Sirous M, Daryani NE, Eshraghi S, Akbari B, Shirazi MH. Assessing the role of the RND efflux pump in metronidazole resistance of Helicobacter pylori by RT-PCR assay. J Infect Dev Ctries. 2011;5(2):88–93. doi:10.3855/jidc.1187. 21389587

[12] Li XZ, Nikaido H. Efflux-mediated drug resistance in bacteria. Drugs. 2004;64(2):159–204. doi:10.2165/00003495-200464020-00004. 14717618

[13] Bina JE, Alm RA, Uria-Nickelsen M, Thomas SR, Trust TJ, Hancock RE. Helicobacter pylori uptake and efflux: basis for intrinsic susceptibility to antibiotics in vitro. Antimicrob Agents Chemother. 2000;44(2):248–254. doi:10.1128/AAC.44.2.248-254.2000. 10639345 PMC89666

[14] Zhang Z, Liu ZQ, Zheng PY, Tang FA, Yang PC. Influence of efflux pump inhibitors on the multidrug resistance of Helicobacter pylori. World J Gastroenterol. 2010;16(10):1279–1284. doi:10.3748/wjg.v16.i10.1279. 20222174 PMC2839183

[15] Lee SM, Kim N, Kwon YH, Nam RH, Kim JM, Park JY, Lee YS, Lee DH. rdxA, frxA, and efflux pump in metronidazole-resistant Helicobacter pylori: their relation to clinical outcomes. J Gastroenterol Hepatol. 2018;33(3):681–688. doi:10.1111/jgh.13906. 28748532

[16] Hirata K, Suzuki H, Nishizawa T, Tsugawa H, Muraoka H, Saito Y, Matsuzaki J, Hibi T. Contribution of efflux pumps to clarithromycin resistance in Helicobacter pylori. J Gastroenterol Hepatol. 2010;25(Suppl 1):S75–S79. doi:10.1111/j.1440-1746.2009.06220.x. 20586871

[17] van Amsterdam K, Bart A, van der Ende A. A Helicobacter pylori TolC efflux pump confers resistance to metronidazole. Antimicrob Agents Chemother. 2005;49(4):1477–1482. doi:10.1128/AAC.49.4.1477-1482.2005. 15793129 PMC1068630

[18] Ge X, Cai Y, Chen Z, Gao S, Geng X, Li Y, Li Y, Jia J, Sun Y. Bifunctional enzyme SpoT is involved in biofilm formation of Helicobacter pylori with multidrug resistance by upregulating efflux pump Hp1174 (gluP). Antimicrob Agents Chemother. 2018;62(11). doi:10.1128/AAC.00957-18. PMC6201075 30181372

